# Epidemiology and treatment status of hepatitis C virus infection among people who have ever injected drugs in Korea: a prospective multicenter cohort study from 2007 to 2019 in comparison with non-PWID

**DOI:** 10.4178/epih.e2021077

**Published:** 2021-10-06

**Authors:** Kyung-Ah Kim, Gwang Hyun Choi, Eun Sun Jang, Young Seok Kim, Youn Jae Lee, In Hee Kim, Sung Bum Cho, Moran Ki, Hwa Young Choi, Dahye Paik, Sook-Hyang Jeong

**Affiliations:** 1 Department of Internal Medicine, Inje University Ilsan Paik Hospital, Goyang, Korea; 2 Department of Internal Medicine, Seoul National University Bundang Hospital, Seoul National University College of Medicine, Seongnam, Korea; 3 Department of Internal Medicine, Soonchunhyang University Bucheon Hospital, Bucheon, Korea; 4 Department of Internal Medicine, Inje University Busan Paik Hospital, Busan, Korea; 5 Department of Internal Medicine, Jeonbuk National University Hospital, Jeonju, Korea; 6 Department of Internal Medicine, Chonnam National University Hwasun Hospital, Hwasun, Korea; 7 Department of Cancer Control and Policy, Graduate School of Cancer Science and Policy, National Cancer Center, Goyang, Korea

**Keywords:** Hepatitis C, Drug users, Epidemiology, Treatment uptake

## Abstract

**OBJECTIVES:**

Injection drug use is a major risk factor for hepatitis C virus (HCV) infection; however, limited data on this topic are available in Korea. Thus, this study aimed to investigate the epidemiological and clinical characteristics, treatment uptake, and outcomes of HCV infection among people who inject drugs (PWID).

**METHODS:**

We used the data from the Korea HCV cohort, which prospectively enrolled patients with HCV infection between 2007 and 2019. Clinical data and results of a questionnaire survey on lifetime risk factors for HCV infection were analyzed according to a self-reported history of injection drug use (PWID vs. non-PWID group).

**RESULTS:**

Among the 2,468 patients, 166 (6.7%) were in the PWID group, which contained younger patients (50.6±8.2 vs. 58.2±13.1 years) and a higher proportion of male (81.9 vs. 48.8%) than the non-PWID group. The distribution of PWID showed significant regional variations. Exposure to other risk factors for HCV infection was different between the groups. The proportion of patients with genotype non-2 infection was higher in the PWID group. Treatment uptake was higher in the PWID group in the interferon era; however, it was comparable between the groups in the direct-acting antiviral era. The rate of sustained virological response did not significantly differ between the groups.

**CONCLUSIONS:**

As of 2019, PWID constituted a minority of HCV-infected people in Korea. The epidemiological characteristics, but not treatment uptake and outcomes, were different between the PWID and non-PWID groups. Therefore, active HCV screening and treatment should be offered to PWID in Korea.

## INTRODUCTION

Hepatitis C virus (HCV) infection affects approximately 70 million people worldwide and is a major cause of liver cirrhosis and hepatocellular carcinoma (HCC) [1]. With the recent advent of highly effective direct-acting antiviral agents (DAAs), HCV infection has become an eradicable disease [2]. However, the status of the “HCV cascade of care” is far from the goal declared by the World Health Organization, which is the elimination of HCV by 2030 by diagnosing, treating, and preventing > 90%, > 80%, and 90% of new infections, respectively [3]. This strategy emphasizes the need for a thorough knowledge of national and subnational epidemiological patterns to screen for hidden HCV infections.

The prevalence of HCV infection varies widely from < 0.5% to nearly 30% across different global regions [4]. The population at high risk of HCV infection includes people who inject drugs (PWID), people with humen immunodeficiency virus (HIV) infection, men who have sex with men, people with a history of transfusion before HCV screening, children born to mothers infected with HCV, and people who have undergone tattooing or piercing. HCV transmission via transfusion and unsafe healthcarerelated injections has declined globally; however, injection drug use has become a major route of transmission in many countries [5,6]. The proportion of PWID among those with HCV infection is 8.5% globally, with a wide variability from 0.9% to 46.6% among countries [7].

The prevalence of anti-HCV antibodies in the Korean adult population has been reported to be 0.60-0.71%, showing an increasing trend with increasing age [8,9]. However, no studies have investigated the proportion, clinical characteristics, and treatment status of PWID among patients with HCV infection in Korea, although the prevalence of anti-HCV antibodies among small groups of PWID was reported to be approximately 50% [10,11]. Thus, we aimed to estimate the proportion of PWID among people with HCV infection from 2007 to 2019 in Korea and investigate their demographic and clinical characteristics, treatment uptake, and outcomes in comparison with non-PWID.

## MATERIALS AND METHODS

### Study design and population

**Figure 1.**
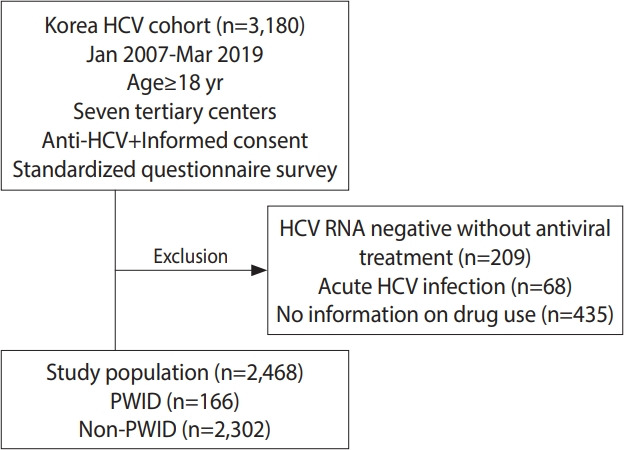
Study population. HCV, hepatitis C virus; PWID, people who inject drugs.

This study analyzed data from the Korea HCV cohort, which prospectively enrolled 3,180 patients from 7 tertiary academic hospitals in Korea between January 2007 and March 2019 (2-center in Seoul enrolled patients from 2007 to 2011, 1 center in Seongnam conducted enrollment from 2007 to 2019, 1 center in Bucheon enrolled patients from 2008 to 2019, 1 center in Busan conducted enrollment from 2010 to 2019, 1 center in Jeonju enrolled patients from 2012 to 2019, and 1 center in Hwasun conducted enrollment from 2013 to 2019). All patients ≥ 18 years of age who were positive for anti-HCV antibody and voluntarily consented to participate in the study were enrolled. A total of 209 patients who were negative for serum HCV RNA with no history of antiviral treatment, 435 patients who did not provide information about injection drug use, and 68 patients with acute infections were excluded from the analysis. The remaining 2,468 patients with viremia who responded to the question about the history of injection drug use were included in this study. Among them, 166 patients were classified as PWID, and the remaining 2,302 patients were classified as non-PWID (Figure 1).

### Data collection and questionnaire survey

After enrollment, trained research coordinators at 7 hospitals interviewed patients using a standardized questionnaire that included questions about their socioeconomic status (age, sex, education level, and occupation), health behaviors (smoking and alcohol drinking), and medical history, including comorbidities such as malignancy, thyroid diseases, psychiatric diseases, diabetes, kidney diseases, cerebrovascular diseases, and cardiovascular diseases. In addition, the survey gathered information on lifetime exposure to possible risk factors for HCV infection, including a history of injection drug use, needlestick injuries, tattooing, piercing, blood transfusions, hemodialysis, hemophilia, dental procedures, endoscopy, acupuncture, surgery, familial history of HCV-related liver disease, residing with HCV carriers, and number of sexual partners. However, information on the initiation, duration, or type of injection drug use was not included in the questionnaire.

Data about laboratory parameters including anti-HCV antibody, serum HCV RNA, hepatitis B virus (HBV) surface antigen, anti-HBV surface antibody, creatinine and alpha-fetoprotein levels, HCV genotype, complete blood count, and liver biochemical variables were collected from patients’ medical records upon enrollment. Data of the results of imaging studies such as abdominal ultrasonography or computed tomography and the results of liver pathology or transient elastography were also collected if available. Detailed information about antiviral treatments, such as the therapeutic regimen, treatment period, and treatment response, was obtained; the antiviral treatment was determined at the discretion of the attending physicians. These data were entered into the established electronic case report form on the homepage of the Korea Disease Control and Prevention Agency, Korea HCV cohort study.

On initial enrollment, the subjects were classified into 3 groups: chronic hepatitis, liver cirrhosis, and HCC. The diagnostic criteria for liver cirrhosis were based on histological findings or at least 1 clinical sign of portal hypertension, such as cirrhotic features on radiological imaging, a platelet count < 100,000/mm^3^, the presence of ascites, gastroesophageal varices, or hepatic encephalopathy. HCC was diagnosed based on histological findings or typical imaging characteristics, as defined by the Korean Liver Cancer Study Group guidelines [12].

The patients were prospectively followed up every 3-12 months at physicians’ discretion. If patients were lost to follow-up for > 6-12 months, the research coordinator at the associated hospital contacted them via phone call to confirm their clinical status and encouraged a follow-up visit [13].

### Statistical analysis

Demographic and clinical characteristics, exposure to risk factors, and antiviral treatment were descriptively analyzed and compared according to their history of injection drug use. Health behaviors and risk factors were compared between PWID and nonPWID who were matched by age (± 5 years), sex, and enrolled year (± 5 years) at a 1:2 ratio considering age and sex differences in lifestyle factors. Treatment uptake and outcomes were analyzed before and after 2015, when DAAs were first approved for HCV treatment in Korea. Multivariable analysis was performed using logistic regression analysis to evaluate factors significantly associated with treatment uptake in the univariable analysis, including injection drug use, for all patients.

Continuous variables, expressed as mean± standard deviation, were compared using the Student t-test. Categorical variables, expressed as absolute and relative frequencies, were compared using the chi-square test. Statistical analyses were performed using IBM SPSS version 25 (IBM Corp., Armonk, NY, USA). All p-values were 2-sided, and the threshold for statistical significance was set at p-value < 0.05.

### Ethics statement

The study protocol was approved by the institutional review board of each hospital, and written informed consent was provided by each enrolled patient before inclusion in the cohort.

## RESULTS

### Epidemiological characteristics of people who inject drugs with hepatitis C virus infection

PWID accounted for 6.7% (166/2,468) of the subjects with HCV infection in Korea. The proportion of PWID among people with HCV infection showed a wide range across geographic regions, from 0.0% in Hwasun to 14.6% in Busan, as well as 9.0% in Seoul, 5.8% in Seongnam, 5.6% in Bucheon, and 0.6% in Jeonju. The overall proportion of PWID was 7.3% (107/14,66) before 2015 and 5.9% (59/1,002) from 2015 onwards (p=0.169). The proportion in Busan was 13.5% (41/304) before 2015 and 15.7% (50/319) from 2015 onwards (p= 0.440). In the PWID group, the patients were younger (50.6± 8.2 vs. 58.2± 13.1 years, p< 0.001) and the proportion of male was higher (81.9 vs. 48.8%, p< 0.001) than in the non-PWID group (Table 1). The PWID group had a higher level of education than the non-PWID group; however, the difference was insignificant after adjusting for age and sex (data not shown). The duration of follow-up was similar in both groups (4.6± 3.0 vs. 4.8± 3.1 years).

There were several differences in exposure to possible risk factors for HCV infection. In particular, the rate of transfusion before 1995, when HCV testing of donated blood was made mandatory in Korea, was significantly higher in the non-PWID group, especially in male, than in the PWID group. In contrast, other possible risk factors, including multiple sexual partners, incarceration, needlestick injury, tattooing, and commercial shaving, were more frequent in the PWID group than in the non-PWID group. The proportions of patients with piercings, those with a history of acupuncture, dental procedure, endoscopy, and hemodialysis, and those residing with HCV carriers were not significantly different between the groups (Table 1). When age-matched and sex-matched groups were compared, piercing and endoscopy were more frequent in the PWID group than in the non-PWID group.

### Clinical characteristics and comorbidity profile of people who inject drugs with hepatitis C virus infection

The mean duration of HCV infection after diagnosis was 9.5 years and 7.8 years in the PWID and non-PWID groups, respectively, indicating that PWID and non-PWID became aware of their HCV infection in their early 40s and early 50s, respectively (Table 2). The proportion of patients with advanced liver disease, including cirrhosis and HCC, was significantly higher in the non-PWID group than in the PWID group; however, the difference was statistically insignificant after adjusting for age and sex (Table 3). Significant differences in the HCV genotype distribution were noted between the groups. The proportion of those with genotype 2 infection was lower in the PWID group (30.4 vs. 48.6%), whereas the proportions of those with genotype 1 (66.5 vs. 50.4%) and other genotypes, including genotypes 3, 4, and 6 (3.1 vs. 1.0%) were higher in the PWID group than in the non-PWID group. HCV reinfection with different genotypes after a sustained virological response (SVR) was identified in three patients, all of whom had no history of injection drug use. There were no significant differences in laboratory parameters, including liver biochemistry, platelet count, and serum HCV RNA levels, between the PWID and non-PWID groups. Fibrosis markers, such as the aspartate-platelet ratio index and fibrosis-4 index, were significantly higher in the non-PWID group than in the PWID group (Table 2).

Comorbidities, including hypertension, chronic kidney disease, and extrahepatic malignancies, were present in more patients in the non-PWID group than in the PWID group. However, when the age-matched and sex-matched groups were compared, these differences were not significant.

The rate of coinfection with HBV was 2.8% and 2.3% in the nonPWID and PWID groups, respectively. HIV coinfection was extremely rare in both groups (0.3 and 0.0% in the non-PWID and PWID groups, respectively). Coinfection with both viruses did not show a significant difference between the 2 groups.

### Treatment uptake and sustained virological response rate in people who inject drugs

The overall treatment uptake rate was higher in the PWID group than in the non-PWID group (76.5 vs. 68.9%). When the rate of treatment uptake was compared before and after 2015, when DAAs were approved in Korea for the first time, it was significantly higher among patients enrolled before 2015 (interferon era) in the PWID group (77.6% vs 64.5%, p= 0.006) than in the non-PWID group. However, among the patients who were enrolled from 2015 onwards (DAA era), the rate of treatment uptake was not meaningfully different between the groups (74.6% vs 75.1% in the PWID and non-PWID groups, respectively, p= 0.931) (Table 4). In the multivariable analysis, age < 70 years, absence of cirrhosis or HCC, absence of diabetes, absence of extrahepatic malignancy, genotype 1 infection, and a recent diagnosis of HCV infection were associated with treatment uptake (Table 5).

The SVR rate with interferon-based therapy was not significantly different between the PWID and non-PWID groups, regardless of the genotype or analysis method, such as per-protocol (PP) or intention-to-treatment (ITT) analyses (Table 4).

DAA regimens did not significantly differ between the nonPWID and PWID groups; daclatasvir+asunaprevir was most commonly prescribed (42.9%) for genotype 1 infections, followed by elbasvir/grazoprevir (21.0%), ledipasvir/sofosbuvir (18.7%), and glecaprevir/pibrentasvir (7.2%). For genotype 2 infections, sofosbuvir+ribavirin was the most popular regimen (75.8%), followed by glecaprevir/pibrentasvir (20.3%). The overall SVR rate of DAA therapy was not significantly different between the PWID and non-PWID groups in a PP analysis (100 vs. 96.4%, p= 0.115); however, it tended to be lower in the PWID group than in the non-PWID group in an ITT analysis (85.9 vs. 92.1%, p= 0.058).

## DISCUSSION

The proportion of PWID among patients with HCV infections in this study was 6.7%; most PWID were presumed to be former users, not current users, according to the physicians’ opinions. The proportion of PWID in this study is lower than has been estimated in Japan and China (20.9 and 8.5%, respectively). Globally, injection drug use accounts for 8.5% of HCV infections, ranging from 1.5% in the Middle East and North Africa to 30.5% in North America [15].

According to studies conducted in the late 2000s in Korea [10,11], almost half of PWID had hepatitis C viremia; their mean age was 42 years and the proportion of male patients was 89%. Moreover, the HBV–HCV coinfection rate was 4.1%, and HCV–HIV coinfection was not observed among PWID. In our study, the proportion of male was 81.8%, the mean age was 51 years, and HIV coinfection was not observed in PWID; these findings are consistent with those of previous research [10].

Globally, it is estimated that there are 15.6 million PWID aged 15-64 years; 27.9% of them are aged < 25 years, and 20.4% are female, with substantial variation across geographic regions. The global prevalence of HCV infection among PWID is estimated to be 52.3%, and the global HIV and HBV prevalence rates among PWID are 17.8% and 9.1%, respectively, with substantial geographic variations [16].

There is no reliable information about the incidence or prevalence of PWID in Korea because injection drug use is illegal in Korea. According to the statistics of the prosecutor’s office in 2018-2020, which is the only source for estimating the number of reported PWID, the number of criminals using illegal drugs including opioids, psychotropic drugs, and marijuana was 18,050; among them, 48% were aged 20-39 years and 22.3% were female [17]. However, the number of criminals using illegal drugs in 2018 and 2019 was 12,613 and 16,044, respectively, meaning that an alarming increase has taken place during the last 3 years. Therefore, the proportion of PWID among patients with HCV infection might increase in the future, which warrants ongoing monitoring of this group.

The risk factors for HCV infection in Korea include transfusion before 1995, intravenous drug use, multiple sex partners, hemodialysis, needlestick injury, and tattooing [18]. Substantial differences were found in exposure to possible risk factors for HCV infection between the PWID and non-PWID groups. Transfusion before 1995 was more frequent in the non-PWID group than in the PWID group, whereas other risk factors, including piercing, tattooing, needlestick injury, incarceration, and multiple sexual partners, were more frequent in the PWID group than in the non-PWID group. Moreover, a larger proportion of patients underwent endoscopy in the PWID group than in the non-PWID group. These findings suggest that the successful eradication of HCV in PWID could reduce the incidence of new infections due to exposure to other risk factors. Globally, injection drug use accounts for 23% of new HCV infections [1], and treatment of PWID with HCV infections is important as part of the treatment-as-prevention strategy to meet the goal of HCV eradication by 2030 [19].

Advanced liver disease (e.g., cirrhosis and HCC) was more common in the non-PWID group than in the PWID group; however, the difference was not statistically significant after adjusting for age and sex. A recent meta-analysis reported that fibrosis progression was faster in PWID than in non-PWID [20]; however, another study showed that the speed of fibrosis progression was not different between PWID without HIV infections and non-PWID [21]. Since none of the PWID in our study had HIV coinfections, it can be concluded that injection drug use did not affect the severity of liver disease in our study.

Genotype 1 infections were more prevalent in the PWID group than in the non-PWID group; however, a study in 2013 showed that the proportions of genotype 1 and 2 infections were comparable between these groups [10]. The reason for the predominance of genotype 1 in our study is unclear, and further studies are necessary to confirm the HCV genotype distribution in PWID.

The rate of uptake of antiviral treatment among PWID before the DAA era was reported to be low, probably due to poor tolerability and lack of efficacy [22,23], although it has increased with DAA treatment in the United States [24]. In contrast, our study showed that the overall treatment rate was higher among PWID than among non-PWID, especially in the pre-DAA era. In the pre-DAA era, old age and comorbidities might have hindered interferon-based therapy, meaning that the PWID group—with younger age and fewer comorbidities—had a higher likelihood of receiving treatment than the non-PWID group. The multivariable analysis showed that the independent factors associated with treatment uptake were younger age, absence of cirrhosis or HCC, and absence of comorbidities, but not intravenous drug use. These findings suggest that the higher treatment uptake in PWID, especially during interferon era, might have resulted from their younger age and fewer comorbidities compared with non-PWID. In addition, the PWID recruited in this cohort were likely to be highly motivated for HCV treatment because they visited university hospitals.

A recent meta-analysis showed similar rates of SVR, adherence to DAAs, and DAA discontinuation between PWID and non-PWID [25]. In this study, the SVR rate of interferon-based therapy was not significantly different between the PWID and non-PWID groups in both ITT and PP analyses. However, the SVR rate of DAA therapy tended to be lower in the PWID group than in the non-PWID group in the ITT analysis, although no difference between the groups was seen in the PP analysis. These findings may be related to compliance or other reasons, which should be studied further because of the small sample size of the PWID group in this study.

This study has several limitations. First, there was no information about whether the history of injection drug use was old, recent, or current. In addition, information on the types of injection drugs or duration of drug use was not available. Second, PWID were defined based only on self-statements concerning injection drug use. This might have underestimated the prevalence of PWID among people with HCV infection, considering that most injection drugs are illegal in Korea. Third, the study population was recruited from tertiary academic hospitals and might have contained people who were disproportionately motivated to seek antiviral treatment. Therefore, the rates of treatment uptake and compliance do not necessarily reflect the overall status of HCV infection in PWID in Korea. However, this study documented the clinical characteristics and treatment status of PWID with HCV infection in Korea for the first time, and the study findings can be useful as basic data to inform the development of a strategy for HCV elimination in Korea.

In conclusion, as of 2019, PWID comprised a minority (6.7%) of all HCV-infected people in Korea. The epidemiological features of the PWID group were different from those of the non-PWID group. However, the treatment uptake and outcomes were not significantly different between these 2 groups in DAA era. Therefore, considering the global and national increase in the number of PWID and the contribution of PWID to new cases of HCV infection, active screening and treatment should be offered to PWID in Korea.

## Figures and Tables

**Table 1.** Comparison of demographic characteristics and exposure to risk factors for HCV infection according to injection drug use

Characteristics	PWID (N=166)	Non-PWID (N=2,302)	p-value
Age at enrollment, mean±SD (yr)	50.6±8.2	58.2±13.1	<0.001
Male, % (n)	81.9 (136)	48.8 (1,124)	<0.001
Follow-up period, mean±SD (yr)	4.8±3.1	4.6±3.0	0.365
Area of residence, % (n)			<0.001
Seoul	9.0 (31)	91.0 (314)	
Seongnam	5.8 (21)	94.2 (338)	
Bucheon	5.6 (21)	94.4 (353)	
Busan	14.6 (91)	85.4 (532)	
Jeonju	0.6 (2)	99.4 (345)	
Hwasun	0.0 (0)	100 (425)	
Education			
≥High school, % (n/N)	69.9 (109/156)	54.9 (1,270/2,302)	<0.001
Alcohol, % (n/N)			
Male	74.8 (101/135)	72.7 (306/1,121)	0.682
Heavy vs. never or social	30.4 (41/135)	22.4 (251/1,121)	0.041
Female	53.8 (14/26)	34.5 (405/1,175)	0.035
Smoking, % (n/N)			
Male	83.6 (112/136)	69.9 (781/1,117)	<0.001
Female	57.7 (15/27)	14.1 (170/1,164)	<0.001
Transfusion before 1995	5.0 (7/141)	17.8 (396/2,228)	<0.001
Multiple sexual partners^1^	66.7 (94/141)	17.7 (367/2,070)	<0.001
Incarceration	60.8 (101/166)	6.3 (141/2,302)	<0.001
Needle stick injury	57.1 (89/156)	3.7 (78/2,118)	<0.001
Tattoo	69.3 (88/127)	56.0 (859/1,534)	0.004
Piercing	27.6 (43/156)	34.3 (788/2,300)	0.087
Acupuncture	84.5 (136/161)	81.8 (1,875/2,291)	0.401
Dental procedure	96.3 (154/160)	96.0 (2,124/2,213)	0.737
Endoscopy	94.6 (139/147)	91.2 (1,987/2,178)	0.163
Commercial shaving^2^	69.4 (68/98)	31.6 (362/1,147)	0.001
Dialysis	0.0 (0/166)	1.5 (34/2,302)	0.166
Living with HCV carrier	1.8 (3/166)	2.5 (57/2,302)	0.793

HCV, hepatitis C virus; PWID, people who inject drugs; SD, standard deviation.

1 Number of partners ≥4.

2 This question was asked since September 2014.

**Table 2.** Comparison of HCV infection status and comorbidities according to injection drug use

Variables	PWID (n=166)	Non-PWID (N=2,302)	p-value
Duration of HCV infection (yr)	9.5±13.7	7.8±12.2	0.087
Severity of liver disease at time of enrollment, % (n)			0.020
Chronic hepatitis	71.2 (121)	62.2 (1,472)	
Liver cirrhosis	20.6 (35)	22.8 (540)	
HCC	5.9 (10)	12.3 (290)	
Baseline laboratory findings			
Serum HCV RNA level (log IU/mL)	5.3±1.9	5.1±1.7	0.211
Platelet count (x1,000/μL)	163.4±58.1	165.5±69.4	0.657
Albumin (g/dL)	4.1±0.4	4.1±0.5	0.139
AST (IU/L)	71.2±55.0	65.0±65.7	0.235
ALT (IU/L)	77.8±68.2	65.3±92.8	0.090
PT INR	1.07±0.15	1.08±0.17	0.860
Total bilirubin (mg/dL)	0.92±0.55	0.96±2.72	0.858
APRI (>1.0), % (n/N)	10.9 (18/165)	17.4 (394/2,258)	0.031
FIB-4 at entry (>3.25), % (n/N)	31.5 (52/165)	42.4 (958/2,262)	0.006
HCV genotype, % (n/N)			<0.001
1	66.5 (107/161)	50.4 (1,092/2,166)	
2	30.4 (49/161)	48.6 (1,052/2,166)	
Others	3.1 (5/161)	1.0 (22/2,166)	
3/4/6/mixed	2/0/3/0	14/2/5/1	
Reinfection of HCV, n	0	3	
Comorbidities, % (n/N)			
Hypertension	19.3 (32/166)	27.1 (624/2,302)	0.027
Diabetes	23.5 (39/166)	19.0 (446/2,366)	0.159
Cancer (except HCC)	2.4 (4/166)	10.0 (231/2,302)	<0.001
Cardiovascular disease	1.2 (2/166)	2.7 (62/2,302)	0.244
Cerebrovascular disease	1.8 (3/166)	2.1 (48/2,302)	0.808
Chronic kidney disease	0.0 (0/166)	1.9 (44/2,302)	0.071
Hemodialysis	0.0 (0/166)	1.5 (34/2,302)	0.166
Psychiatric diseases	5.4 (9/166)	4.4 (101/2,302)	0.533
HBsAg positivity	2.3 (2/86)	2.8 (39/1,383)	0.787
HIV coinfection	0.0 (0/43)	0.3 (2/784)	1.000

PWID, people who inject drugs; HCV, hepatitis C virus; HCC, hepatocellular carcinoma; AST, aspartate transferase; ALT, alanine transferase; PT, prothrombin time; INR, international normalized ratio; APRI, aspartate transferase to platelet ratio index; FIB-4, fibrosis-4; HBsAg, hepatitis B virus surface antigen; HIV, human immunodeficiency virus.

**Table 3.** Comparison of liver disease status, comorbidities, and exposure to risk factors for HCV infection between PWID and age-and sex-matched non-PWID

Variables	PWID (N=166)	Matched non-PWID^1^ (N=332)	p-value
Age (yr)	50.6±8.2	51.9±9.5	0.120
Sex (M/F), n	136/30	272/60	
Education ≥high school, % (n/N)	69.9 (107/153)	78.0 (255/327)	0.056
Severity of liver disease, % (n)			0.750
Chronic hepatitis	72.9 (121)	69.9 (232)	
Liver cirrhosis	21.1 (35)	24.1 (80)	
HCC	6.0 (10)	6.0 (20)	
HCV genotype, % (n/N)			0.026
1	66.5 (107/161)	57.0 (184/323)	
2	30.4 (49/161)	41.8 (135/323)	
Others	3.1 (5/161)	1.2 (4/323)	
3/4/6/mixed	2/0/3/0	3/0/0/1	
Comorbidities			
Hypertension	19.3 (32/166)	19.0 (63/332)	1.000
Diabetes	23.5 (39/166)	16.3 (540/332)	0.051
Cancer (except HCC)	2.7 (4/166)	6.3 (21/332)	0.080
Cardiovascular disease	1.2 (2/166)	1.5 (5/332)	1.000
Cerebrovascular disease	1.8 (3/166)	3.0 (10/332)	0.558
Chronic kidney disease	0.0 (0/166)	2.4 (8/332)	0.057
Psychiatric diseases	5.4 (9/166)	3.9 (13/332)	0.185
Risk factors, % (n/N)			
Transfusion before 1995	5.0 (7/141)	20.0 (64/320)	<0.001
Multiple sexual partners^2^	66.7 (94/141)	34.1 (100/293)	<0.001
Incarceration	60.8 (101/166)	13.3 (44/332)	<0.001
Needle stick injury	57.1 (89/156)	3.4 (10/295)	<0.001
Tattoo	69.3 (88/127)	43.9 (72/164)	<0.001
Piercing	27.6 (43/156)	17.2 (57/332)	0.008
Acupuncture	84.5 (136/161)	79.1 (261/330)	0.155
Dental procedure	96.3 (154/160)	93.8 (301/321)	0.257
Endoscopy	94.6 (139/147)	86.9 (271/312)	0.013
Commercial shaving^3^	69.4 (68/98)	43.9 (74/132)	<0.001
Hemodialysis	0.0 (0/166)	1.8 (6/332)	0.480
Living with HCV carrier	1.8 (3/166)	1.8 (6/332)	1.000

HCV, hepatitis C virus; PWID, people who inject drugs; M, male; F, female; HCC, hepatocellular carcinoma; HBsAg, hepatitis B virus surface antigen.

1 Matched for age (±5 years), sex and enrolled year (±5 years).

2 Number of partners ≥4.

3 This question was asked since September 2014.

**Table 4.** HCV treatment uptake and outcomes according to injection drug use

Variables	PWID (n=166)	Non-PWID (n=2,302)	p-value
Age at IFN treatment	49.2±7.5	54.1±10.8	<0.001
Age at DAA treatment	53.4±7.4	60.7±11.3	<0.001
Treatment uptake rate, % (n/N)	76.5 (127/166)	68.9 (1,585/2,302)	0.039
Enrollment from 2008 to 2014 (IFN era)	77.6 (83/107)	64.5 (877/1,359)	0.006
Enrollment from 2015 to 2019 (DAA era)	74.6 (44/59)	75.1 (708/943)	0.931
Therapeutic regimen, n			
IFN-based therapy	73	815	
DAA	78	940	
Naïve	54	770	
IFN experienced	24	166	
DAA experienced	0	4	
DAA, regimen			
Daclatasvir+asunaprevir	27	235	
Ledipasvir/sofosbuvir	12	107	
Sofosbuvir+ribavirin	13	292	
Glecaprevir/pibrentasvir	11	111	
Elvasvir/grazoprevir	7	126	
OPr+D^1^	2	36	
Others^2^	6	33	
SVR, % (n/N)			
IFN-based therapy (ITT analysis)			
Genotype 1	43.8 (21/48)	46.5 (177/381)	0.723
Genotype 2	65.0 (13/20)	74.5 (286/384)	0.496
Others^3^	60.0 (3/5)	78.0 (39/50)	0.582
IFN-based therapy (PP analysis)			
Genotype 1	44.7 (21/47)	48.4 (178/368)	0.647
Genotype 2	65.0 (13/20)	77.3 (286/370)	0.205
Others^3^	60.0 (3/5)	79.5 (39/49)	0.306
DAA (ITT analysis)	85.9 (67/78)	92.1 (862/936)	0.058
Genotype 1	87.5 (49/56)	91.4 (507/555)	0.337
Genotype 2	78.9 (15/19)	93.0 (346/372)	0.025
Others^3^	100 (3/3)	100 (9/9)	
DAA (PP analysis)	100 (67/67)	96.4 (862/894)	0.115
Genotype 1	100 (49/49)	96.4 (507/526)	0.176
Genotype 2	100 (15/15)	96.4 (346/359)	0.453
Others^3^	100 (3/3)	100 (9/9)	

PWID, people who inject drugs; IFN, interferon; DAA, direct anti-viral agents; SVR, sustained virological response; ITT, intention-to treatment; PP, per protocol.

1 Ombitasvir/paritaprevir/ritonavir+dasabuvir.

2 Includes boceprevir+pegylated interferon, simeprevir+sofosbuvir, dacla-tasvir+sofosbuvir, sofosbuvir/velpatasvir, and unknown regimens.

3 Includes genotype 3, 4, 6 and no information for genotype.

**Table 5.** Multivariable analysis for factors associated with treatment uptake

Factors	Univariable analysis	Multivariable analysis
Age (≥70 vs. <70)	0.27 (0.22, 0.34)	0.36 (0.28, 0.45)
Sex (male vs. female)	1.16 (0.98, 1.38)	1.01 (0.83, 1.23)
Year of enrollment (by 1-year increment)	1.07 (1.04, 1.10)	1.12 (1.09, 1.15)
Liver disease severity		
Chronic hepatitis	1.00 (reference)	1.00 (reference)
Cirrhosis	0.68 (0.56, 0.84)	0.72 (0.58, 0.91)
HCC	0.20 (0.15, 0.26)	0.26 (0.19, 0.65)
Extrahepatic malignancy	0.70 (0.53, 0.92)	0.65 (0.48, 0.90)
Diabetes	0.67 (0.54, 0.82)	0.78 (0.62, 0.98)
Intravenous drug use	1.48 (1.02, 2.14)	1.08 (0.72, 1.64)
Genotype (1 vs. 2)	0.72 (0.60, 0.87)	0.75 (0.62, 0.92)

Values are presented as odds ratio (95% confidence interval). HCC, hepatocellular carcinoma.
